# Prevalence of trypanosomes associated with drug resistance in Shimba Hills, Kwale County, Kenya

**DOI:** 10.1186/s13104-020-05077-3

**Published:** 2020-04-29

**Authors:** Benard W. Kulohoma, Sarah A. O. Wamwenje, Ibrahim I. Wangwe, Nicodemus Masila, Caroline K. Mirieri, Lillian Wambua

**Affiliations:** 1 Centre for Biotechnology and Bioinformatics, University of Nairobi, P.O. Box 30197, Nairobi, 00100 Kenya; 2 International Centre of Insect Physiology and Ecology, Nairobi, Kenya; 3 Kenya Tsetse and Trypanosomiasis Eradication Council (KENTTEC), Kwale County, Kenya; 4 Directorate of Veterinary Services, Vector Regulatory and Zoological Services, Makindu, Kenya; 5 School of Biological Sciences, University of Nairobi, Nairobi, Kenya; 6 Present Address: International Livestock Research Institute, Nairobi, Kenya

**Keywords:** Tsetse flies, *TbAT1*, Trypanosomiasis, Prevalence, Drug resistance, Infectivity

## Abstract

**Objective:**

Animal African trypanosomiasis (AAT) is a life-threatening vector-borne disease, caused by trypanosome parasites, which are principally transmitted by tsetse flies. In Kenya, the prevalence of drug-resistant trypanosomes in endemic regions remains poorly understood. The objective of this study was to establish AAT point prevalence, drug susceptibility of associated trypanosomes, and measure infectivity by multiple AAT mammalian hosts to tsetse flies in Shimba hills, a resource-poor region with high bovine trypanosomiasis prevalence and morbidity rates at the coast of Kenya. We collected tsetse flies using traps (1 Ngu and 2 biconical), and then sorted them on sex and species. Trypanosomes present in tsetse flies were detected by first extracting all genomic DNA, and then performing PCR reactions with established primers of the internal transcribed spacer regions. Polymorphisms associated with trypanocide resistance in the *TbAT1* gene were also detected by performing PCR reactions with established primers.

**Results:**

Our findings suggest low trypanosome prevalence (3.7%), low trypanocide resistance, and low infectivity by multiple mammalian hosts to tsetse flies in Shimba hills. We conclude that enhanced surveillance is crucial for informing disease management practices that help prevent the spread of drug-resistant trypanosomiasis.

## Introduction

Animal African trypanosomiasis (AAT) is a life-threatening disease in cattle, sheep and goats caused by trypanosome parasites (*Trypanosoma congolense, Trypanosoma brucei, Trypanosoma vivax, T. simiae and T. suis*) [1]. Tsetse flies, the principal vectors of trypanosomes, transmit parasites by feeding on blood meals of infected hosts host. Repeated treatment with the same trypanocides to manage trypanosomiasis exerts selection pressure at the drug targeted trypanosome genes. Consequently, trypanosomes acquire mutations that confer trypanocidal drug resistance. Tsetse flies occur widely with varying densities across and within countries in sub-Saharan Africa [2, 3]. In Kenya, AAT endemic regions are infested with up to 1000 tsetse flies per square kilometre [1, 4, 5]. These vectors can be found in 38 of the 47 counties occupying approximately 138 000 km^2^ (23% of the country) [1, 4, 5]. Previous studies have found high bovine trypanosomiasis prevalence (33.9%) and morbidity rate (29.1%) in Shimba Hills, Kwale county [6, 7]. Shimba Hills is a rural resource-poor setting at the coast of Kenya that is adjacent to a nature reserve [6, 7]. Shimba Hills has high interaction of domestic animals, humans and wild animals, for example buffaloes and wildebeests, which increases trypanosome transmission rates via tsetse fly bites.

### Main text

Chemotherapy and chemoprophylaxis are the main strategies for AAT management. Approximately 70 million doses are procured annually by farmers in sub-Saharan Africa [8, 9]. Nonetheless, there is growing concern over the rapidly developing, widespread, multi-drug resistance to the few available trypanocidal drug classes (isometamidium, homidium and diminazene). This has led to poor treatment outcomes [9–11]. High prevalence of drug resistant trypanosomes has been reported even after stopping drug use in endemic areas [12]. Thus, it is crucial to characterize the distribution of drug resistant trypanosomes to optimize strategies to control or eliminate trypanosomiasis. However, trypanocide resistance levels vary widely across eastern and southern Africa. High severity has been identified in coastal regions of Kenya and Tanzania, where elevated multi-trypanocide resistance is associated with a history of increased drug usage, in contrast to western Kenya and Zambia where only single-drug resistance has been reported [11]. We evaluated AAT trypanosome point prevalence, drug susceptibility of associated trypanosomes, and measured infectivity by multiple AAT mammalian hosts to tsetse flies in Shimba hills, an endemic region at the coast of Kenya, to understand changes in trypanosomiasis epidemiology. In a cross-sectional study, in October 2015, we collected tsetse flies, and established infection with trypanosome species by PCR. We examined polymorphisms in the trypanosome adenosine transporter (*TbAT1*) gene, which encodes for a nucleoside transporter implicated in resistance to diminazene aceturate, melarsporol and other trypanocides. DNA sequences of the *TbAT1* gene generated from infected tsetse in this collection were compared to drug-sensitive reference sequences of *T. b. rhodensiense*, *T. brucei brucei* and *T. b. gambiense* [13–16] to detect mutations.

## Methods

### Study site and sample collection

We collected tsetse flies after 24 h using three traps (1 Ngu and 2 biconical) in Shimba Hills (latitude−4.174°S and longitude 39.4602 °E) (Additional file 1), Kwale county, Kenya, in October 2015 [1, 17]. These stationary traps were deployed at a distance of 200 m apart. The trapped tsetse flies retrieved from the traps were sorted by sex and species, and stored in separate eppendorf tubes with unique identifiers in liquid nitrogen for shipment to the laboratory, after which they were stored at − 80 °C.

### DNA extraction and genotyping

We extracted all genomic DNA present in the tsetse flies. Briefly, each tsetse fly sample was separately homogenized by adding 0.3 g of glass beads in an eppendorf tube, followed by rotation on a homogenizer at 5000 rpm for 45 s. Samples were lysed by adding 300 μl of lysis buffer (10 mM TRIS (pH 0.8), 0.5% SDS, 5 mM EDTA), followed by incubation at 65 °C for 15 min. Proteins were precipitated by adding 100 μl of 8 M ammonium acetate, 1 mM EDTA; vortexing for 30 s; placing on ice for 5 min; and centrifugation at 16,400× g for 5 min. The supernatant was added to a separate tube with 100 μl of 100% isopropanol, inverted 10 times, and then centrifuged at 16,400 x g for 5 min. The supernatant was discarded and 300 μl of absolute ethanol added to the remaining pellet, and then gently inverted 5 times, followed by centrifugation at 16,400× g for 1 min.

Ethanol was pipetted off and 300 μl of ice cold 70% ethanol was added to the remaining pellet, followed by centrifugation at 16,400× g for 5 min at 4 °C. The supernatant was discarded and the previous step repeated once. Ethanol was pipetted off and the tubes inverted on tissue paper and left to dry overnight in a sterile laminar flow hood. The samples were then hydrated with 50 μl of nuclease free water, and allowed to incubate at 65 °C for 1 h. Hydrated DNA concentration and purity was determined using a nanodrop ND-2000 instrument (Thermo Fischer Scientific, UK). The DNA concentration was then diluted to 50 ng/μl for all samples. PCR reactions were performed to detect presence of trypanosome DNA from the extracted genomic DNA. Established universal primers, and MyTaq™ DNA polymerase kit (Bioline, UK) were used, following the manufacturer’s instructions. Trypanosomes present in tsetse flies were specifically detected using previously established universal primers (ITS1-CF: 5′ CCGGAAGTTCACCGATATTG 3′, melting temperature 58.4 °C; and ITS1-BR: 5′ TTGCTGCGTTCTTCAACGAA 3′, melting temperature 56.4 °C) with high specificity to detect and distinguish trypanosomes by the length of the internal transcribed spacer (ITS) gene region of rDNA [18, 19]. Established primers for drug susceptibility trypanosome adenosine transporter (*TbAT1*) gene were used (TbAT/P2-F: 5′ GAAATCCCCGTCTTTTCTCAC 3′, melting temperature 59.4 °C; and TbAT/P2-R: 5′ ATGTGCTGACCCATTTTCCTT 3′, melting temperature 57.4 °C) to identify polymorphisms associated with drug resistance [20]. Amplicon quality was assessed by electrophoresis in 1.5% agarose gel. Absence of trypanosomes was confirmed by performing a second repeat confirmatory PCR. Three independent *TbAT1* amplification reactions were performed for each trypanosome positive sample, the PCR products were pooled, and purified (QIAquick kit, Qiagen, Basel, Switzerland). Sanger DNA sequencing was outsourced to Macrogen (Seoul, South Korea). Quality control and editing of nucleic sequence trace files were performed using CLC genomics workbench software version 9.5.3 (CLC bio, QIAGEN, Redwood City, USA). Multiple sequence alignments were performed using MUSCLE [21]. We compared gene sequences at the *TbAT1* gene that encodes a nucleoside transporter associated with resistance to multiple trypanocides generated from *Trypanosoma b. brucei* (11/14) isolates in our study (GenBank accession numbers MK751607- MK751617) to those of established drug-sensitive reference sequences of *T. brucei* [16], to detect polymorphisms associated with trypanocide resistance.

## Results

**Fig. 1:**
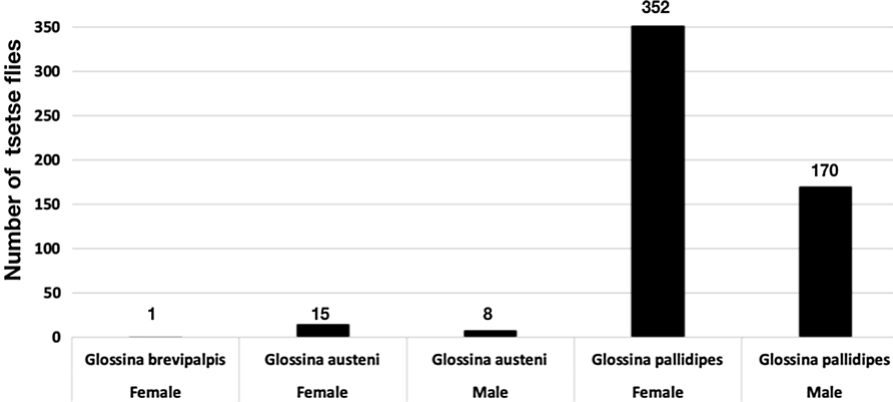
The distribution of all vectors collected. Shows the sex of each tsetse fly species collected

The study included 546 tsetse flies collected after 24 h from three (1 Ngu and 2 Biconical) traps deployed 200 m apart in Shimba hills in October 2015. We observed a significant difference (Fischers exact test *p *< 0.004) in the number of insects trapped between the Ngu and a single Biconical trap (Table 1). A disproportionate number of female tsetse flies was collected (67.4%, 368/546). The largest proportion (95.6%, 522/546) of trapped tsetse flies were of the savanna species *Glossina pallidipes* (Fig. 1).

**Table 1:** The distribution of vectors caught per trap

	Ngu	Biconical 1	Biconical 2
Male	57	48	73
Female	160	66	142
% of insects caught	39.7% (n = 217)	20.9% (n = 114)	39.4% (n = 215)
	*	*	–
	**	–	**
	–	***	***

The Ngu trap performed significantly better than Biconical trap 1 (Fishers exact test: *p < 0.004; **p = 0.009; ***p = 0.15). The asterisks show the traps compared for each of the three p-values

We detected trypanosome infection using PCR in 3.7% (20/546) of the *Glossina pallidipes* species from the entire tsetse flies collected (Table 2). We identified three closely related trypanosome species, which cause animal African trypanosomiasis, from the tsetse flies collected: *Trypanosoma congolense* (15%, 3/20)*, Trypanosoma brucei brucei* (70%, 14/20) and *Trypanosoma vivax* (15%, 3/20). A single mixed infection with *Trypanosoma brucei brucei* and *Trypanosoma vivax* was also detected. Trypanosomes were detected in more female compared to male tsetse flies (Table 2).

**Table 2:** Trypanosome infected tsetse flies

Sample ID	Vector species	Vector sex	Trap used	*T. b. brucei*	*T. congolense*	*T. vivax*
GpFB-10	*Glossina pallidipes*	Female	Biconical 2	√	–	–
GpFB-11	*Glossina pallidipes*	Female	Biconical 2	√	–	–
GpFB-35	*Glossina pallidipes*	Female	Biconical 2	–	–	√
GpFB-44	*Glossina pallidipes*	Female	Biconical 2	–	√	–
GpFB-45	*Glossina pallidipes*	Female	Biconical 2	–	–	√
GpFB-47	*Glossina pallidipes*	Female	Biconical 2	√	–	–
GpFB-49	*Glossina pallidipes*	Female	Biconical 2	√	–	–
GpFB-59	*Glossina pallidipes*	Female	Biconical 2	√	–	–
GpFB-63	*Glossina pallidipes*	Female	Biconical 2	√	–	–
GpFB-65	*Glossina pallidipes*	Female	Biconical 2	√	–	–
GpFB-67	*Glossina pallidipes*	Female	Biconical 2	√	–	–
GpFB-75	*Glossina pallidipes*	Female	Biconical 2	√	–	√
GpFB-79	*Glossina pallidipes*	Female	Biconical 2	√	–	–
GpFB-88	*Glossina pallidipes*	Female	Biconical 2	√	–	–
GpFN-61	*Glossina pallidipes*	Female	Ngu		√	–
GpFN-75	*Glossina pallidipes*	Female	Ngu		√	–
GpMB1-14	*Glossina pallidipes*	Male	Biconical 1	√	–	–
GpMB1-24	*Glossina pallidipes*	Male	Biconical 1	√	–	–
GpMB1-29	*Glossina pallidipes*	Male	Biconical 1	√	–	–
GpFB1-22	*Glossina pallidipes*	Female	Biconical 1	√	–	–

The table shows vector species, sex, and trypanosome infection

Comparative analysis of amino acid sequences from our study with those from drug-sensitive reference strains did not highlight any polymorphisms previously reported to be associated with drug resistance (Additional file 2). The amino acid residues at polymorphic sites were all similar to those in the drug-sensitive reference strains.

## Discussion

Our study suggests that despite an abundance of tsetse flies in Shimba Hills, there is a low number of trypanosome-infected flies, and no evidence of polymorphisms associated with trypanocide non-susceptibility in *T. b. brucei*; the predominant parasite species. Our findings on trypanosome infections in tsetse flies (3.7%) are similar to those of others (3.4%) from the adjacent country, Maasai Steppe, northern Tanzania, during the same period (June 2015 to February 2016) [22]. They also support previous findings of 5.8% trypanosome infection rate of *G. pallidipes*, in Mtito Andei Division, Makueni County, a neighbouring County in Kenya between April and May 2012 [23]. This finding supports a recent report from Nigeria, showing a decline of AAT prevalence over the last six decades [24].

It remains unclear whether differences in trap performance can be attributed to varying behaviour and response by tsetse flies [25]. Both vector sexes feed on blood and transmit trypanosomes, however, we collected more female tsetse flies, and detected more trypanosomes in female tsetse flies. Tsetse fly sex influences susceptibility to trypanosomes infection, and male insects have been shown to be vulnerable to infection with *T. brucei* compared to females [26]. Tsetse flies do not recover after trypanosome infection during their lifespan, and this may explain the lower number of male flies, which are more predisposed to infection [26, 27].

The predominant vector species was *Glossina pallidipes*, which transmits trypanosomes associated with both human and animal disease. We detected *Trypanosoma brucei brucei, Trypanosoma vivax* and *Trypanosoma congolense*. *T. congolense* causes disease in cattle, camels, horses, dogs, sheep, goats, and pigs [28]*. T. vivax* causes disease in cattle, sheep, goats, and horses [28]. *T. brucei brucei* causes disease in horses, camels, dogs, sheep, goats, cattle, and pigs [28]. All three trypanosome species also cause disease to several groups of wild mammals, for example buffaloes and wildebeests [28]. Interestingly, we did not detect *Trypanosoma brucei gambiense* and *Trypanosoma brucei rhodesiense* associated with human disease in the tsetse flies collected, consistent with recent reports of plummeting cases of human African trypanosomiasis due to increased trypanosomiasis control [29]. Our findings are consistent with a previous report on the absence of human-infective trypanosomes in northern Tanzania [22]. The relatively high prevalence of *Trypanosoma brucei brucei* supports previous findings of its predominance in endemic locales after chemotherapeutic elimination of *Trypanosoma vivax* and *Trypanosoma congolense* [30]. This difference in trypanosome prevalence was thought to be due to seasonality, with an observation of *Trypanosoma brucei* abundance at the beginning of the wet season (October). However, seasonal parasite variations are not significant and parasite chronicity, and the biology and epidemiology of transmitting tsetse flies are thought to drive recurrent infections across seasons [22].

Drug resistance is due to accumulation of mutations at transporter genes implicated in trypanocide uptake, resulting in non-susceptibility and a progressive increase in parasite fitness cost that may diminish the population to resistant trypanosomes [1, 16]. We did not detect polymorphisms associated with trypanocide resistance at the *TbAT1* gene. This suggests that drug resistant trypanosomes have been replaced by susceptible strains with a lower fitness cost, or such polymorphisms if present, occur at low levels of frequency undetectable by the sampling size of the present study. Similarly, reduction or change in trypanocides invoking selection pressure may trigger clonal expansion of susceptible trypanosomes with lower fitness cost that out-compete the resistant parasites, reversing resistance to drugs that were in long use [31]. The decline in disease burden could be attributed to renewed efforts to eradicate tsetse and trypanosomiasis by national and multinational implementation of a series novel and existing of interventions [1, 2, 17, 32]. However, the ecologically diverse and geographically widespread areas infested by tsetse flies still pose a challenge in resource allocation decisions and implementation of interventions available. Our findings provide insight on the changing epidemiology of trypanosomiasis that is useful in disease management.

## Limitations

The limitation in our study was using a cross-sectional study design, and we anticipate that future studies with a longitudinal study design over more extended periods of time and including more sampling sites would provide more insight on the dynamics of trypanocide non-susceptibility. Our study was limited by a small sample size and only sampled once, which would significantly reduce statistical power. We had a limited number of traps (n = 3) available. We also only examined polymorphisms associated with resistance at the *TbAT1* gene that encodes a nucleoside transporter, and is associated with resistance to multiple trypanocides. We suggest that subsequent studies that investigate other additional polymorphisms at different gene loci associated with trypanocide resistance, combined with laboratory bioassays would improve understanding of trypanosome drug non-susceptibility.

## Supplementary information

**Additional file 1.** Map showing the study sites (Kizibe and Mbegani) in Kwale County, Kenya.

**Additional file 2.** Aligned amino acid sequences of the TbAT1 genes.

## Data Availability

All materials and data used to perform this study are available in the main text. The 11 TbAT1 gene sequences generated in this study are accessible in GenBank with the accession numbers MK751607- MK751617.

## Abbreviations

×g: Times the Earths gravitational force
AAT: Animal African trypanosomiasis
Km^2^: Square kilometre
%: Percent
^o^S: Degrees south
^o^E: Degrees East
^o^C: Degrees Celsius
rpm: Revolutions per minute
M: Mole
mM: Micromole
ng: Nanogram
μl: Microliter
EDTA: Ethylenediaminetetraacetic acid
PCR: Polymerase chain reaction
SDS: Sodium dodecyl sulfate
TRIS: 2-Amino-2-(hydroxymethyl)-1,3-propanediol
