# Supplementary figures and images for: Prevalence of trypanosomes associated with drug resistance in Shimba Hills, Kwale County, Kenya
Source: BMC Res Notes. 2020 Apr 29;13:234. doi: 10.1186/s13104-020-05077-3 (PMC7191804; doi:10.1186/s13104-020-05077-3)

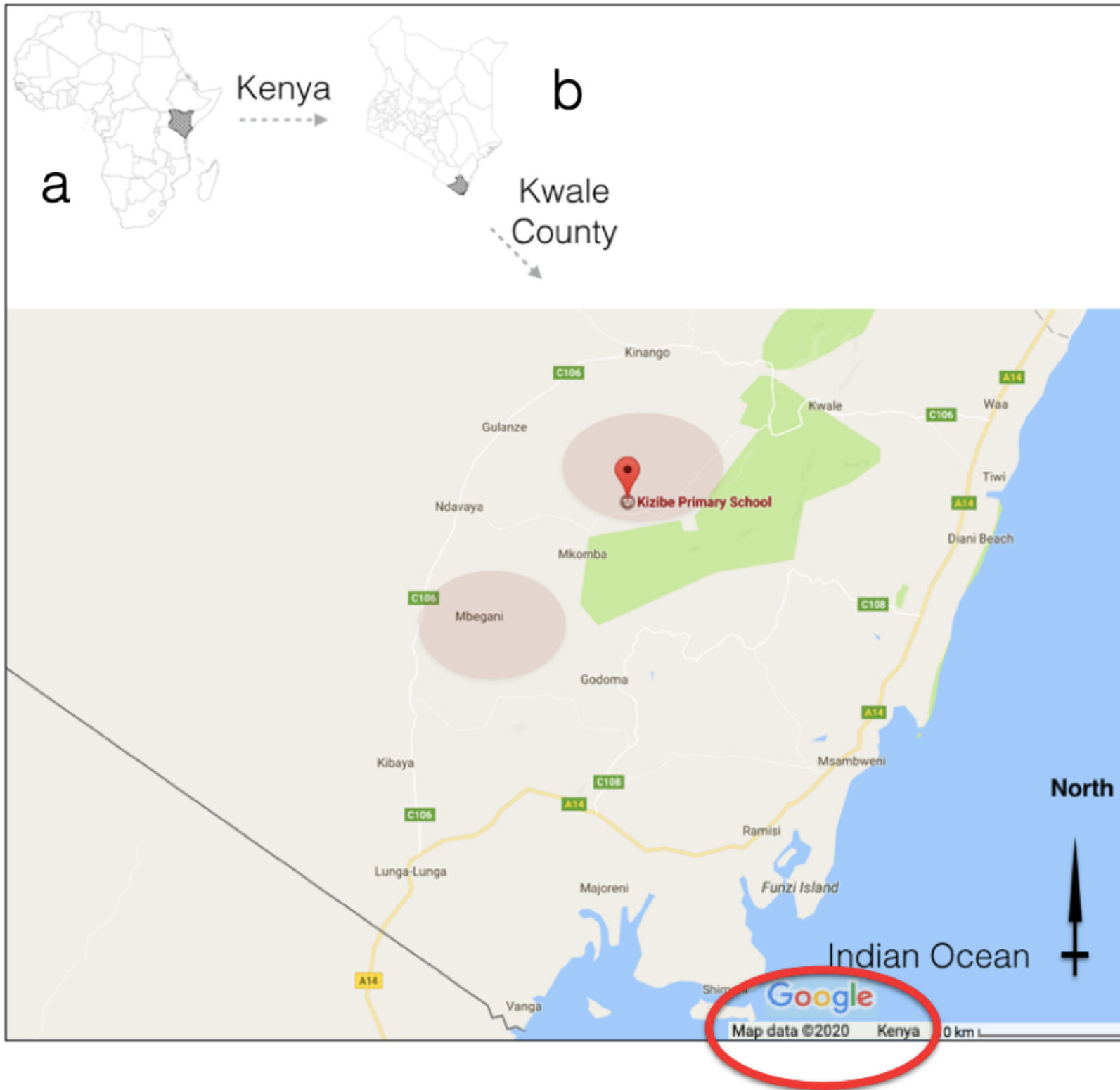

C

Supplement: Supplementary file 1 — Additional file 1. Map showing the study sites (Kizibe and Mbegani) in Kwale County, Kenya. [file 13104_2020_5077_MOESM1_ESM.pdf]
